# Analgen

**Published:** 1893-09-02

**Authors:** 


### NEW DRUGS AND PREPARATIONS.

[We should be glad to receive, at our Office, 428, Strand, London, W.C., from the manufacturers, specimens of all new preparations and drugs which may be brought out from time to time.]

### ANALGEN.

Several of the recent synthetical remedies have amply proved their claim to be classed among the efficient analgesics, and in spheres of action where formerly opium stood almost alone as the only drug upon which we could rely, it has now many formidable rivals claiming our notice. Many practitioners have expressed their satisfaction at the results obtained from the use of phenazonum and phenactine in certain painful affections, chiefly neuralgias and pain symptomatic of certain diseases accompanied by fever, such as epidemic influenza.

It is often very difficult to say whether a pain will be relieved by these remedies, and a feeling of uncertainty as to the result not infrequently makes us hesitate to run the risk of wasting time and wearing out the patience of our clients. On this account we were prepared to give a hearty welcome to a drug which, bearing the name of analgen, should surely prove that certain means of relief that suffering humanity desires.

We are not prepared to maintain that our personal experience with analgen justifies any positive opinion as to its value compared with other analgesics, but we are bound to confess that, while it is by no means a "universal pain-killer," it has afforded marked relief in a few of our cases, and apparently in many patients treated with this drug in Germany.

Analgen, or Ortho-aethoxy-ana-monobenzoylamidoquinoline, we are informed by Messrs. Christy, is prepared by Vis by treating oxyquinoline with caustic soda and ethylbromide, producing aethoxyquinoline. By the action of nitric acid this is converted into aethoxynitroquinoline, which is then reduced to the amido combination. This is treated with benzoylchloride and soda in aqueous solution, and thus yields to the product in question, which after repeated recrystalization is obtained in its pure state as a crystalline white powder insoluble in water, readily soluble in hot and sparingly soluble in cold water.

Case L. W., age 27, a married lady of decidedly neurotic temperment, subject to intense neuralgic headaches since girlhood, which formerly occurred periodically. More recently headaches, if slightly less severe, have been much more frequent, and sufficiently intense to render her unable to engage in any household duties whilst they lasted. Has also suffered from intense neuralgia in the lumbar region. Has generally obtained speedy relief to the cephalalgia from considerable doses of phenazonum and bromide of potassium, but only in such doses as rendered her incapable of activity for some time, for which reason she was sometimes felt obliged to forego its use. Took ten grains of analgen in cachet, followed by a second dose in half an hour, from which she obtained rapid relief from headache. The same pleasing result was obtained from similar doses during an attack of backache. No disagreeable symptoms were observable, nor was there any depression afterwards.

The family history showed a strong gouty tendency, and although no true gout had displayed itself in this case, the headaches were probably lithæmic.

Case A. S., widow, age 55, a thin, spare woman, recovering from acute rheumatism, successfully relieved by salicylate of sodium. Complained of intense sciatica in the left hip. This was an old trouble, and was undoubtedly connected with the rheumatic diathesis. Took five grains of analgen every hour till four doses were taken. No relief. Then returned to the salicylate of soda, the second doze of twenty grains relieving the pain.

This patient had a very feeble heart and was very weakly, but no untoward symptoms were observed. The analgen was repeated on another occasion, but without any result, good or bad.

Other observers appear to have obtained better results. Thus Dr. Keberlet reports the case of (1) Mrs. H., about 45, has for several years suffered from sciatica in the right leg. In the autumn of 1892 a relapse again took place, more severe than any before, so that the patient was unable to leave the bed and could not sleep. All the remedies tried, morphia, quinine, antifebrine, antipyrine, faradization, massage, were of no avail. Then he prescribed cachets of ½ gramme analgen. At once very considerable relief was experienced, and after 10 to 15 catchets the patient was perfectly free from pain and could walk without difficulty. She took one cachet three times daily. This effect of analgen was quite striking, as it clearly occurred simultaneously with the administration of the remedy.

(2) A man of about 70 had suffered for a fortnight from severe sciatic pains in the left leg, he could not get up or move, and had no peace day or night. After 10 cachets of ½ gramme analgen there was considerable decrease in pain and the movements of the leg were freer; after a further 10 cachets the recovery was complete.

In another case under our care, F. C., age 42, a lady, who had had a double öophorectomy, performed some years previously without relief to the lumbar pain and general neurasthenia, from which she had been a sufferers for years. This patient was extremely susceptible to most drugs, but unfortunately could not be relieved except by morphine in such doses that the after-effects always made her hesitate to resort to its use. Phenacetine, phenazonum, and the bromides had one and all been tried on several occasions without the slightest benefit.

Ten grains of analgen gave partial relief, but, she declared, produced symptoms similar to those resulting from the use of morphine, without relieving her pains to the same extent. The small dose of five grains gave no result. In this case the analgen was discontinued, but the patient was a very difficult subject to prescribe for, and the pain complained of was probably unduly exaggerated.

In another case we administered analgen to a male patient aged 38, complaining of pains in the heels, ankle joints, and shoulders, with a history of gonorrhœa with subsequent gleet and stricture. He had been treated with mistura guaiaci (Throat Hosp. Pharm.), taken three times daily without mitigation of the pain. After two days we prescribed ten grains of analgen every four hours, but after six doses he experienced no relief from pain. He was then told to take ten grains every hour for three doses with the result that the pain was very greatly relieved.

This experience hardly confirms the favourable report of Dr. Treupel, assistant at the medical dispensary at Breslau, who reports as follows: 1. Rheumarthritis gonorrhoica. Five grammes analgen in doses of one-half gramme considerably lessened the pain in the wrist and fore-arm; the affected parts (middle of the hand, wrist, and half the fore-arm) were kept at rest and treated with damp warm compresses, and the large swelling abated.

After a further five grammes analgen the pain became less, the wrist was more pliable and admitted of massage, which hitherto on account of the great pain had been impossible. In this case other anti-neuralgics, also acid salicylic, as well as the application of blisters, had only brought temporary relief. The case was already in an advanced stage when it came under treatment. It was not cured by us, as sufficient attention could not be given to the primary complaint a gonorrhoical genital affection. The patient (a female) was transferred to Neisser's hospital."

Miss L., age 24, three years ago had suffered from intense occipital neuralgia, without any obvious cause. Her physician prescribed bromides, phenazonum, caffein, and various tonics, but it lasted more or less for six weeks. No remedy seemed to relieve the headache. A few weeks ago the headache came back again, and as the old prescriptions had been tried and failed, we gave her cachets of analgen eight grains. One to be taken and followed in an hour by a second. No relief. She was also taking nervine tonics, and her general health improved. She has only been under observation one week.

M. Althause, Ph.D., Barmen, writing to the proprietor, reports: "Since the winter 1882 I have suffered from neuralgic headache which with slight variations returns half-yearly. The suffering always comes on after a previous general cold, and commences with pains in the right half of the head. As a rule, these pains pass from the supraorbital nerve over the right eye, and then pass over the whole of the head. At first they are continuous, but as the illness increases the throb corresponds with the beats of the pulse. Severe pressure often comes on in the larynx in connection with slight retching. The pain commences in the morning, then increases gradually until noon, when it becomes so severe and intolerable that one would think the head was going to burst. Towards evening the pain slowly decreases. Any movement, even the very slightest, causes sudden and severe increase of pain. The attack lasts 14 days, when the pain disappears as slowly as it came. This unbearable condition and the slight prospect of relief has at last made me contemplate undergoing a severe operation. After hearing of analgen, I took during an unusually severe attack of neuralgia two grammes, and in the course of two days I was completely relieved. An hour after taking it a reaction set in, the pain first, getting weaker then a pleasant, bright feeling in the head followed. While taking analgen I have noticed no bad effects."

*(To be continued.)*

